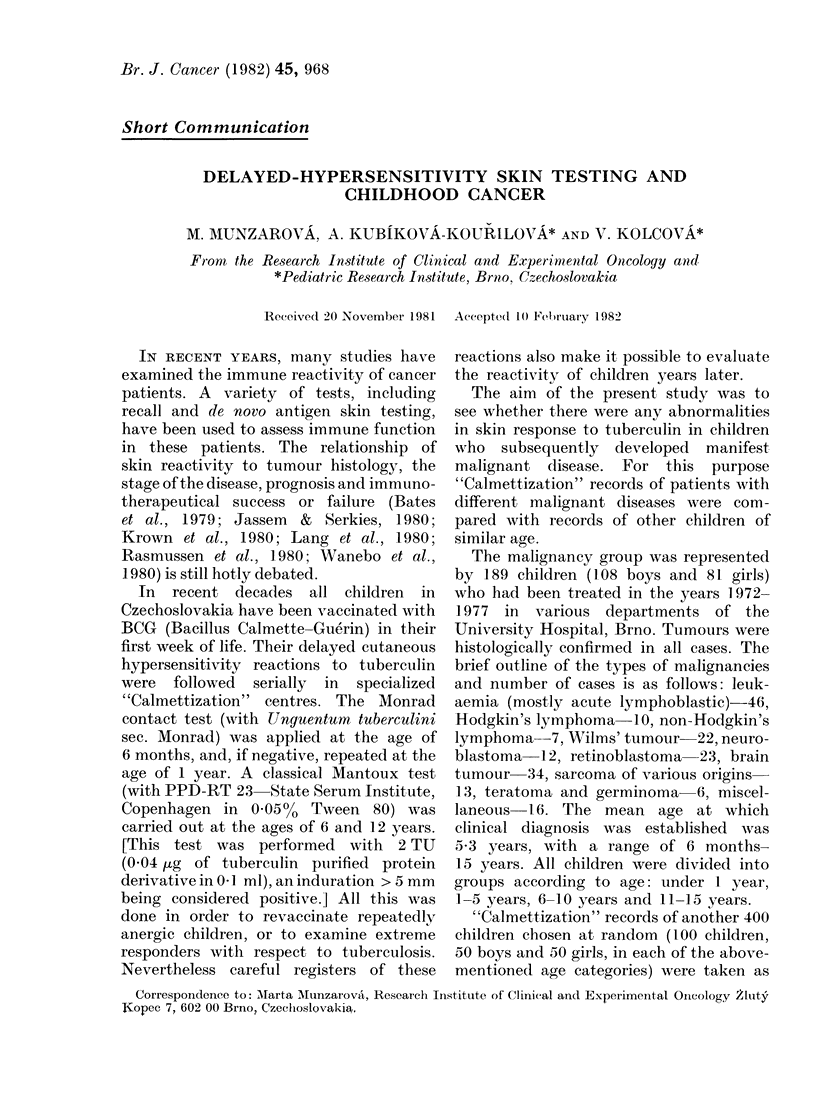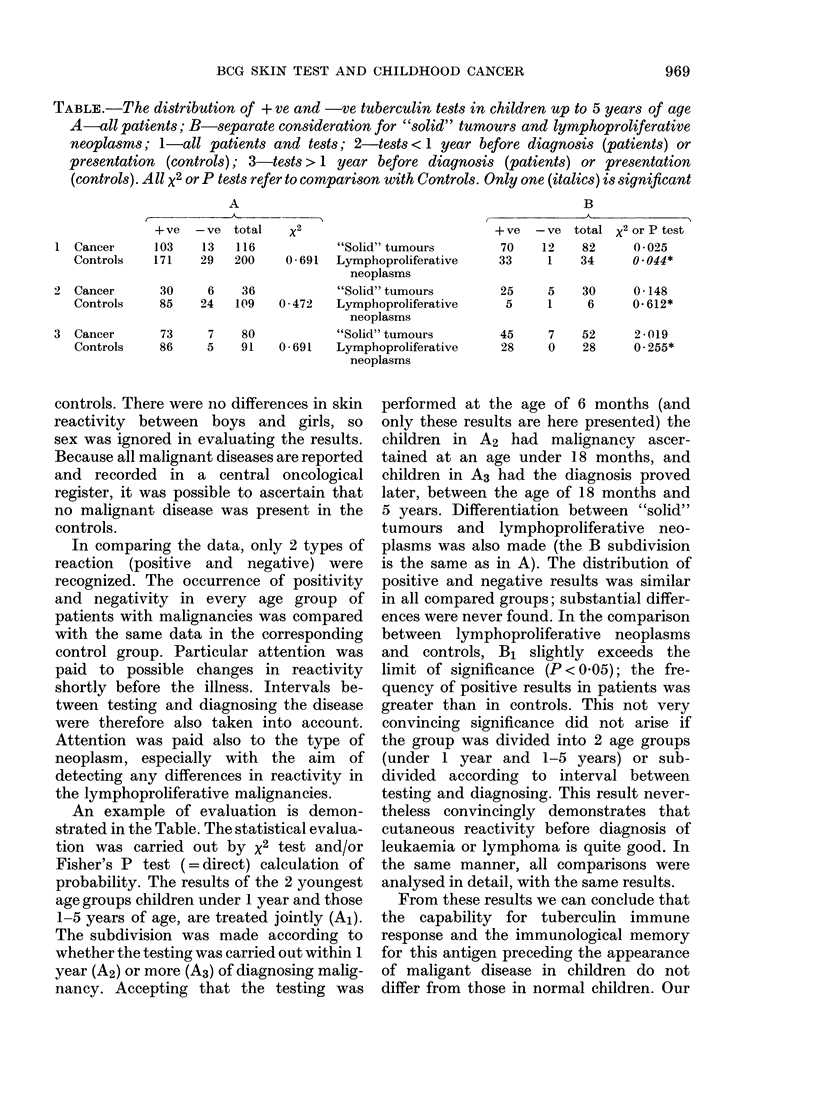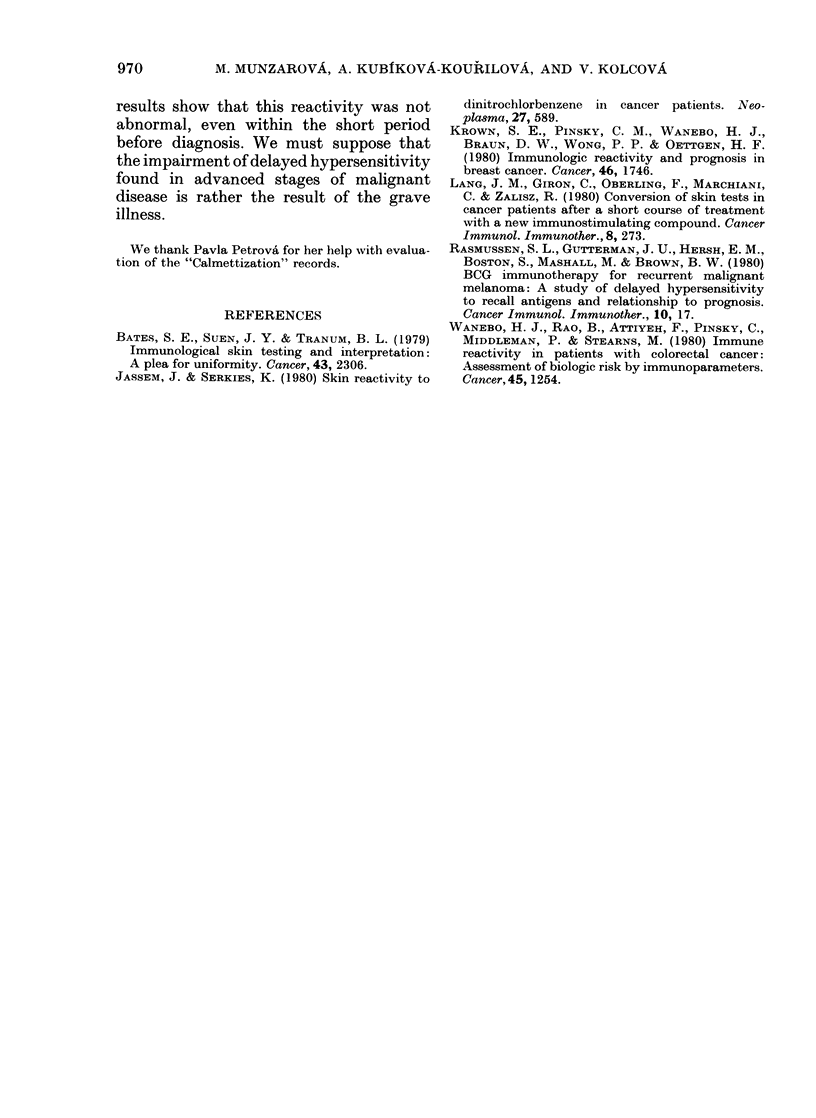# Delayed-hypersensitivity skin testing and childhood cancer.

**DOI:** 10.1038/bjc.1982.150

**Published:** 1982-06

**Authors:** M. Munzarová, A. Kubíková-Kourilová, V. Kolcová


*Br. J. Cancer* (1982) **45**, 968

***Short Communication***

### DELAYED-HYPERSENSITIVITY SKIN TESTING AND CHILDHOOD CANCER

M. MUNZAROVÁ, A. KUBÍKOVÁ-KOUŘILOVÁ* AND V. KOLCOVÁ*

*From the Research Institute of Clinical and Experimental Oncology and *Pediatric Research Institute, Brno, Czechoslovakia*

Received 20 November 1981 Accepted 10 February 1982

IN RECENT YEARS, many studies have examined the immune reactivity of cancer patients. A variety of tests, including recall and *de novo* antigen skin testing, have been used to assess immune function in these patients. The relationship of skin reactivity to tumour histology, the stage of the disease, prognosis and immunotherapeutical success or failure (Bates *et al.*, 1979; Jassem & Serkies, 1980; Krown *et al.*, 1980; Lang *et al.*, 1980; Rasmussen *et al.*, 1980; Wanebo *et al.*, 1980) is still hotly debated.

In recent decades all children in Czechoslovakia have been vaccinated with BCG (Bacillus Calmette–Guèrin) in their first week of life. Their delayed cutaneous hypersensitivity reactions to tuberculin were followed serially in specialized "Calmettization" centres. The Monrad contact test (with *Unguentum tuberculini* sec. Monrad) was applied at the age of 6 months, and, if negative, repeated at the age of 1 year. A classical Mantoux test (with PPD-RT 23—State Serum Institute, Copenhagen in 0·05% Tween 80) was carried out at the ages of 6 and 12 years. [This test was performed with 2 TU (0·04 $\mu$g of tuberculin purified protein derivative in 0·1 ml), an induration $>5$ mm being considered positive.] All this was done in order to revaccinate repeatedly anergic children, or to examine extreme responders with respect to tuberculosis. Nevertheless careful registers of these reactions also make it possible to evaluate the reactivity of children years later.

The aim of the present study was to see whether there were any abnormalities in skin response to tuberculin in children who subsequently developed manifest malignant disease. For this purpose "Calmettization" records of patients with different malignant diseases were compared with records of other children of similar age.

The malignancy group was represented by 189 children (108 boys and 81 girls) who had been treated in the years 1972–1977 in various departments of the University Hospital, Brno. Tumours were histologically confirmed in all cases. The brief outline of the types of malignancies and number of cases is as follows: leukaemia (mostly acute lymphoblastic)—46, Hodgkin's lymphoma—10, non-Hodgkin's lymphoma—7, Wilms' tumour—22, neuroblastoma—12, retinoblastoma—23, brain tumour—34, sarcoma of various origins—13, teratoma and germinoma—6, miscellaneous—16. The mean age at which clinical diagnosis was established was 5·3 years, with a range of 6 months–15 years. All children were divided into groups according to age: under 1 year, 1–5 years, 6–10 years and 11–15 years.

"Calmettization" records of another 400 children chosen at random (100 children, 50 boys and 50 girls, in each of the above-mentioned age categories) were taken as

Correspondence to: Marta Munzarová, Research Institute of Clinical and Experimental Oncology Žlutý Kopec 7, 602 00 Brno, Czechoslovakia.

TABLE.—*The distribution of +ve and —ve tuberculin tests in children up to 5 years of age A—all patients; B—separate consideration for "solid" tumours and lymphoproliferative neoplasms; 1—all patients and tests; 2—tests < 1 year before diagnosis (patients) or presentation (controls); 3—tests > 1 year before diagnosis (patients) or presentation (controls). All $\chi^2$ or P tests refer to comparison with Controls. Only one (italics) is significant*

| | | A | | | | | B | | | |
|---|---|---|---|---|---|---|---|---|---|---|
| | | +ve | −ve | total | $\chi^2$ | | +ve | −ve | total | $\chi^2$ or P test |
| 1 | Cancer | 103 | 13 | 116 | | "Solid" tumours | 70 | 12 | 82 | 0·025 |
| | Controls | 171 | 29 | 200 | 0·691 | Lymphoproliferative neoplasms | 33 | 1 | 34 | *0·044** |
| 2 | Cancer | 30 | 6 | 36 | | "Solid" tumours | 25 | 5 | 30 | 0·148 |
| | Controls | 85 | 24 | 109 | 0·472 | Lymphoproliferative neoplasms | 5 | 1 | 6 | 0·612* |
| 3 | Cancer | 73 | 7 | 80 | | "Solid" tumours | 45 | 7 | 52 | 2·019 |
| | Controls | 86 | 5 | 91 | 0·691 | Lymphoproliferative neoplasms | 28 | 0 | 28 | 0·255* |

controls. There were no differences in skin reactivity between boys and girls, so sex was ignored in evaluating the results. Because all malignant diseases are reported and recorded in a central oncological register, it was possible to ascertain that no malignant disease was present in the controls.

In comparing the data, only 2 types of reaction (positive and negative) were recognized. The occurrence of positivity and negativity in every age group of patients with malignancies was compared with the same data in the corresponding control group. Particular attention was paid to possible changes in reactivity shortly before the illness. Intervals between testing and diagnosing the disease were therefore also taken into account. Attention was paid also to the type of neoplasm, especially with the aim of detecting any differences in reactivity in the lymphoproliferative malignancies.

An example of evaluation is demonstrated in the Table. The statistical evaluation was carried out by $\chi^2$ test and/or Fisher's P test (=direct) calculation of probability. The results of the 2 youngest age groups children under 1 year and those 1–5 years of age, are treated jointly ($A_1$). The subdivision was made according to whether the testing was carried out within 1 year ($A_2$) or more ($A_3$) of diagnosing malignancy. Accepting that the testing was performed at the age of 6 months (and only these results are here presented) the children in $A_2$ had malignancy ascertained at an age under 18 months, and children in $A_3$ had the diagnosis proved later, between the age of 18 months and 5 years. Differentiation between "solid" tumours and lymphoproliferative neoplasms was also made (the B subdivision is the same as in A). The distribution of positive and negative results was similar in all compared groups; substantial differences were never found. In the comparison between lymphoproliferative neoplasms and controls, $B_1$ slightly exceeds the limit of significance ($P < 0{\cdot}05$); the frequency of positive results in patients was greater than in controls. This not very convincing significance did not arise if the group was divided into 2 age groups (under 1 year and 1–5 years) or subdivided according to interval between testing and diagnosing. This result nevertheless convincingly demonstrates that cutaneous reactivity before diagnosis of leukaemia or lymphoma is quite good. In the same manner, all comparisons were analysed in detail, with the same results.

From these results we can conclude that the capability for tuberculin immune response and the immunological memory for this antigen preceding the appearance of maligant disease in children do not differ from those in normal children. Our results show that this reactivity was not abnormal, even within the short period before diagnosis. We must suppose that the impairment of delayed hypersensitivity found in advanced stages of malignant disease is rather the result of the grave illness.

We thank Pavla Petrová for her help with evaluation of the "Calmettization" records.

### REFERENCES

Bates, S. E., Suen, J. Y. & Tranum, B. L. (1979) Immunological skin testing and interpretation: A plea for uniformity. *Cancer*, **43**, 2306.

Jassem, J. & Serkies, K. (1980) Skin reactivity to dinitrochlorbenzene in cancer patients. *Neoplasma*, **27**, 589.

Krown, S. E., Pinsky, C. M., Wanebo, H. J., Braun, D. W., Wong, P. P. & Oettgen, H. F. (1980) Immunologic reactivity and prognosis in breast cancer. *Cancer*, **46**, 1746.

Lang, J. M., Giron, C., Oberling, F., Marchiani, C. & Zalisz, R. (1980) Conversion of skin tests in cancer patients after a short course of treatment with a new immunostimulating compound. *Cancer Immunol. Immunother.*, **8**, 273.

Rasmussen, S. L., Gutterman, J. U., Hersh, E. M., Boston, S., Mashall, M. & Brown, B. W. (1980) BCG immunotherapy for recurrent malignant melanoma: A study of delayed hypersensitivity to recall antigens and relationship to prognosis. *Cancer Immunol. Immunother.*, **10**, 17.

Wanebo, H. J., Rao, B., Attiyeh, F., Pinsky, C., Middleman, P. & Stearns, M. (1980) Immune reactivity in patients with colorectal cancer: Assessment of biologic risk by immunoparameters. *Cancer*, **45**, 1254.